# Correlation of Two-Minute and Six-Minute Walk Tests With Spirometric Indices in Patients With Severe Chronic Obstructive Pulmonary Disease at a Selected Tertiary Care Hospital in Puducherry

**DOI:** 10.7759/cureus.74619

**Published:** 2024-11-27

**Authors:** Antonious Selvam, Seran Durai, Dishan Y, Rajalakshmi M, Praveen Radhakrishnan

**Affiliations:** 1 Pulmonary Medicine, Pondicherry Institute of Medical Sciences, Puducherry, IND; 2 Pulmonary Medicine, Sri Venkateshwara Medical College and Hospital, Puducherry, IND; 3 Pulmonary Medicine, Dr. Somervell Memorial CSI Medical College and Hospital, Trivandrum, IND; 4 Community Medicine, Sri Manakula Vinayagar Medical College and Hospital, Puducherry, IND; 5 Pulmonary Medicine, Sri Manakula Vinayagar Medical College and Hospital, Puducherry, IND

**Keywords:** 2-minute walk test, 6-minute walk test, chronic obstructive pulmonary disease(copd), sp02, spirometry

## Abstract

Background

Chronic obstructive pulmonary disease (COPD) is a progressive respiratory condition characterised by airflow limitation and reduced exercise capacity. The Six-Minute Walk Test (6MWT) and Two-Minute Walk Test (2MWT) are commonly used to assess functional exercise capacity in COPD patients. This study aims to evaluate the correlation between the distance covered in the 2MWT and 6MWT with spirometric indices (such as Forced Expiratory Volume in 1 second (FEV₁), Forced Vital Capacity (FVC), and FEV₁/FVC) in COPD patients.

Materials and methods

This cross-sectional study involved 50 severe and very severe COPD patients diagnosed according to Global Initiative for Chronic Obstructive Lung Disease (GOLD) criteria. Systematic random sampling was adopted to select the study participants. Each patient underwent spirometry to measure FEV₁, FVC, and FEV₁/FVC ratio. Following spirometry, both the 2MWT and 6MWT were conducted on separate days under standardised conditions.

Results

The average distance covered by the patients with very severe obstruction in 6MWT and 2MWT was 148±5.0 and 113.60±4.4. Patients with severe obstruction in 6MWT and 2MWT covered an average distance of 163.10±12.4 and 125.73±11.8, respectively. The mean FEV₁/FVC ratio and the mean FEV₁ % in 6MWT were 38.4 and 30.6 with a significant p-value of <0.001. The mean FEV₁/FVC ratio and the mean FEV₁ % in 2MWT were 32 and 29, respectively, with a significant p-value of <0.003. Both 2MWT and 6MWT distances were significantly correlated with spirometric indices, particularly FEV₁ and FEV₁/FVC ratio.

Conclusion

We found that there was no significant difference among spirometric indices between the two groups. Hence, 2MWT can be used as a reliable prognostic screening tool as it poses an advantage like decreasing the exhaustion for COPD patients as they find it more difficult to complete the 6MWT, resulting in increased heart rate and easy fatiguability.

## Introduction

Dyspnea, coughing, sputum production, and exacerbations are among the chronic respiratory symptoms of chronic obstructive pulmonary disease (COPD), a heterogeneous lung condition caused by abnormalities of the airways (bronchitis, bronchiolitis) and/or alveoli (emphysema) that result in persistent, frequently progressive airflow obstruction [1]. About 90% of deaths from COPD occur in low- and middle-income countries (LMIC), making it the third most common cause of mortality at the moment. Early diagnosis and therapy are essential to lessen flare-ups and decrease the progression of symptoms [2]. Due to ongoing exposure to COPD risk factors and population aging, the burden of COPD is expected to rise globally over the next several decades. Globally, COPD is the primary cause of chronic morbidity and mortality. Many people with COPD or its consequences die young and suffer from the illness for years [3]. While the incidence of tobacco use is frequently closely linked to the prevalence of COPD, major risk factors for COPD in many nations include indoor, outdoor, and workplace air pollution from burning wood and other biomass fuels [4]. It is estimated that COPD causes over three million deaths worldwide each year [5]. Over 5.4 million people will die from COPD and related diseases each year by 2060 as a result of rising smoking rates in LMICs and aging populations in high-income nations [6]. Exercise capacity, a crucial component of COPD management, is typically assessed using field walk tests that provide insights into disease severity and patient prognosis. The Six-Minute Walk Test (6MWT) is a study that offers prognostic, treatment response, and functional data useful for treating patients with heart and respiratory conditions with its simplicity and consistency provide a combined picture of the musculoskeletal and cardiopulmonary responses to exercise, making it a popular tool [7]. Since the 6MWT is directly correlated with low quality of life indices, respiratory and functional impairment, and survival, it has been recognized as a key indicator of disease severity for patients with COPD, which also shows the clinical reaction to chronic respiratory conditions and procedures like lung volume reduction surgery [8]. The average Six-Minute Walk Distance (6MWD) for chronic lung illness is between 300 and 400 meters [8]. The risk of aggravation, hospitalization, and death is inversely connected with a 6MWD of 350 meters in people with COPD [9]. 

However, for patients with severe COPD, including those with comorbidities like cardiovascular disease, neuromuscular disorders, or frailty, completing the 6MWT can be challenging due to fatigue and dyspnea. As a result, the Two-Minute Walk Test (2MWT), which is shorter in duration, has been suggested as a potentially useful alternative for assessing functional capacity in patients who cannot perform the full 6MWT [10]. Although less commonly used, the 2MWT may offer advantages in severe cases by reducing physical exhaustion while providing valuable prognostic information​ [10]. Hence, 2MWT, which has a shorter duration, would possibly be an effective alternative tool for the assessment of prognosis in severe COPD patients. Our study aimed to assess the correlation of 6MWT and 2MWT with spirometric indices among severe and very severe COPD patients.

## Materials and methods

Study area and setting

The present study was conducted by the Department of Respiratory Medicine, Sri Manakula Vinayagar Medical College and Hospital, Puducherry. The target participants were severe and very severe COPD patients attending the outpatient department (OPD). 

Study design

A hospital-based analytical cross-sectional study was conducted at the Department of Respiratory Medicine at a tertiary care hospital in Puducherry. The study aimed to evaluate the correlation between 2MWT and 6MWT distances and spirometric indices (forced expiratory volume (FEV₁), forced vital capacity (FVC), and FEV₁/FVC ratio) among patients with severe and very severe COPD as per Global Initiative for Chronic Obstructive Lung Disease (GOLD) criteria and Modified Medical Research Council (MMRC) breathlessness guidelines.

Study participants

All the patients with severe and very severe COPD attending the Respiratory Medicine OPD were included in the study. Ethical principles such as respect for the persons, beneficence, justice, and ensuring confidentiality were adhered to, throughout the study. Informed written consent was obtained from all participants.

Study duration

The study was conducted for 18 months from 26.02.2021 till 25.02.2022 after obtaining Institutional Ethical clearance. The study was approved by the Sri Manakula Vinayagar Medical College and Hospital Ethics Committee (EC/35/2021).

Sample Size

The sample size was calculated using N master software, taking into consideration the sample size of the previous study [11], which was done on the association of 2MWT and 6MWT. It was calculated to be 12; since it was too small, it was inflated to 50. 

Sampling

Systematic random sampling was adopted to select the study participants. To achieve the desired sample size, all the patients attending the respiratory medicine department with severe and very severe COPD were included in the study after spirometric evaluation. Patients visiting OPD on Monday, Wednesday, and Friday were chosen for a 2MWT followed by a 6MWT on the next day, and patients visiting on Tuesday, Thursday, and Saturday were made to perform a 6MWT followed by a 2MWT on the next day.

Inclusion and exclusion criteria

Patients over 18 years old with severe and very severe COPD (GOLD criteria) who provided informed consent were included. Exclusion criteria were patients with active pulmonary tuberculosis, other chronic lung diseases, systemic diseases, or those unwilling to participate.

Data collection procedure

Data was collected by the Principal Investigator (PI) using a pretested semi-structured questionnaire (Appendix), which comprised sociodemographic details, clinical history, anthropometric measurements, and severity of COPD assessed using GOLD criteria. The procedure of spirometry was explained to the patient in simple language. It was done using a computerized vitalograph series using WinspiroPRO 5.8 pneumotach, following the principles of spirometry in a sitting position. Spirometric indices (such as FEV₁, FVC, and FEV₁/FVC) were evaluated in patients.

The tests were carried out in the fixed hour of the day (10.00-14.00 hrs) to minimize diurnal variation, and the walk test was also performed in the same hours of the day to avoid intraday variability. All walk tests were conducted by the trained PI on the same 30-meter track and time of day, following the guidelines of the American Thoracic Society (ATS). Patients were instructed to remain on regular medication schedules and were allowed to walk at their own pace. The distance walked by the patients, as well as pre-and post-test saturation and vitals, was recorded and analyzed at the end of two minutes and six minutes.

Statistical analysis

All data were entered in Excel (Microsoft, Redmond, WA, USA) and analyzed in SPSS v26.0 (IBM Corp., Armonk, NY, USA). Descriptive statistics, expressed as mean ± standard deviation, captured baseline characteristics such as distance walked and spirometric values (FEV₁, FVC, FEV₁/FVC) and frequency with percentage for categorical variables. Comparative analyses were done using independent t-tests for continuous variables. Pearson correlation analysis demonstrated a strong, statistically significant correlation between the distance covered in both walk tests and spirometric indices. All tests were considered statistically significant if the p-value was less than 0.05.

## Results

In this study, 50 patients with severe and very severe COPD participated; the study population primarily consisted of older adults, with 34 (68%) aged over 60 years and 16 (32%) below 60 years. Males were in the majority, comprising 42 (84%), while females accounted for eight (16%). Regarding smoking status, 37 (74%) were smokers, reflecting the strong association between smoking and COPD, while 13 (26%) were non-smokers. In terms of disease severity based on GOLD staging, 26 (52%) had severe obstruction (Grade III), and 24 (48%) had very severe obstruction (Grade IV), indicating that nearly half of the participants were in the most advanced stages of COPD (Table 1). 

**Table 1:** Characteristics of Study Participants (n=50) GOLD - Global Initiative for Chronic Obstructive Lung Disease

Characteristics	Frequency	Percentage
Age group		
<60 years	16	32
>60 years	34	68
Gender		
Male	42	84
Female	08	16
Smoking status		
Yes	37	74
No	13	26
GOLD Staging		
Severe obstruction (Grade III)	26	52
Very severe obstruction (Grade IV)	24	48

Table 2 shows distinct differences in clinical parameters measured during the 2MWD and 6MWD among severe COPD patients. The mean oxygen saturation (SpO₂) was slightly higher in the 2MWD (89.42±2.3) compared to the 6MWD (87.42±2.7), suggesting that patients experienced less desaturation during the shorter test. Heart rate measurements also differed, with a lower mean heart rate in the 2MWD (94.51±3.5 beats/min) than in the 6MWD (101.56±3.8 beats/min), indicating a reduced cardiovascular strain in the shorter test. Patients with very severe obstruction (Grade IV) during 6MWD and 2MWD covered a mean distance of 148.10±5.0 and 125.73±11.8, respectively, with a statistically significant p-value of <0.003. 

**Table 2:** Distribution of Clinical Parameters Among the Study Participants (n=50) MWD - Minute Walk Distance, SpO2 - oxygen saturation

Clinical parameters	Mean±SD
SpO_2 _	
6MWD	87.42±2.7
2MWD	89.42±2.3
Heart Rate (beats/min)	
6MWD	101.56±3.8
2MWD	94.51±3.5
Distance Walked (in metres)	
6MWD	148±5.0
2MWD	125.73±11.8

The mean distance covered by the patients in 6MWD with Grade III and Grade IV MMRC was 163.79±13.0 and 156.69 ±11.8, respectively, with a p-value of 0.139, which was not statistically significant. The mean distance covered by the patients in 2MWD with Grade III and Grade IV MMRC was 129.16±8.0 and 117.44±12, respectively, with a statistically significant p-value of <0.001 (Table 3).

**Table 3:** Association of 6MWD and 2MWD With MMRC Grading Among the Study Participants (n=50) MMRC - Modified Medical Research Council, MWD - minute walk distance Independent samples t test *p-value <0.05 is considered significant

Variable	MMRC III (n=24)	MMRC IV (n=26)	p value*
Distance walked (6 minutes)	163.79±13.0	156.69±11.8	0.139
Distance walked (2 minutes)	129.16±8.0	117.44±12.1	<0.001

The mean FEV₁/FVC ratio and the mean FEV₁ % among patients in 6MWT were 38.4 and 30.6, respectively, which was statistically significant with a p-value of <0.001, as shown in Table 4. The mean FEV₁/FVC ratio and the mean FEV₁ % among patients in 2MWT were 38.4 and 30.6, respectively, which was statistically significant with a p-value of <0.003.

**Table 4:** Association of FEV₁/FVC and FEV₁% Among the Study Participants (n=50) FEV₁ - Forced Expiratory Volume in 1 Second, FVC - Forced Vital Capacity, MWD - Minute Walk Distance Independent samples t test *p-value <0.05 is considered significant

Walk Test	DISTANCE (MEAN+SD)	FEV₁/FVC (MEAN+SD)	FEV₁% (MEAN+SD)	P-VALUE*
6MWD	148±5.0	38.4 ±2.0	30.6±2.3.0	<0.001
2MWD	125.73±11.8	32.2±1..8	29.4±1.4	<0.003

Table 5 shows strong correlations between various physiological parameters measured during the 2MWT and 6MWT and COPD severity levels, classified by MMRC grades III and IV. Walk distance was highly correlated with disease severity, with r = 0.80 for MMRC III and r = 0.77 for MMRC IV, indicating that more severe COPD is associated with shorter walking distances. Minimum oxygen saturation also strongly correlated with severity (r = 0.81 in MMRC III and r = 0.83 in MMRC IV), suggesting that oxygen desaturation reflects the extent of respiratory impairment. Maximum heart rate during the tests had a correlation of r = 0.85 in MMRC III and r = 0.76 in MMRC IV, pointing to increased cardiovascular strain in more severe cases. Similarly, the highest breathing frequency was strongly correlated with disease severity (r = 0.75 in MMRC III and r = 0.79 in MMRC IV), as patients in advanced COPD stages required higher respiratory rates to compensate for limited airflow. 

**Table 5:** Correlations Between Two-Minute Walk Test and Six-Minute Walk Test Parameters Among the Study Participants (n = 50). MMRC - Modified Medical Research Council Pearson's correlation test *p-value <0.05 is considered significant

Parameters	MMRC III (n=24) r	MMRC IV (n=26) r
Walk distance	0.80*	0.77*
Minimum oxygen saturation during test	0.81*	0.83*
Maximum heart rate during test	0.85*	0.76*
Highest breathing frequency during test	0.75*	0.79*

## Discussion

The study was carried out among 50 patients with a confirmed diagnosis of COPD as per GOLD guidelines, and they were subjected to 6MWD and 2MWD followed by spirometry on alternate days. The present study assessed the association between the 2MWT and 6MWT distances with spirometric indices in patients with severe and very severe COPD. The findings indicate a statistically significant correlation between the distance walked in both tests and spirometric parameters, specifically FEV₁, FVC, and the FEV₁/FVC ratio. Patients in the severe COPD group (GOLD stage III) consistently walked longer distances in both the 2MWT and 6MWT compared to those in the very severe COPD group (GOLD stage IV), reflecting progressive functional decline with increasing COPD severity. 

Gupta et al. studied the relationship between lung function tests (spirometry), the 6MWT, and breathlessness severity in COPD patients. They found that the distance covered during the 6MWT was closely linked to the severity of dyspnea as measured by the MMRC scale. However, patients with other health issues like heart disease, muscle disorders, or arthritis often found it difficult to complete the 6MWT due to fatigue [11]. This suggests that the 2MWT is a suitable alternative to the 6MWT, especially for patients with severe COPD. Our study addresses including the 2MWT as an alternative assessment, a shorter test with potential advantages for patients with severe COPD. Gloeckl et al. compared the 2MWT and the 6MWT to see how well they detect exercise-related drops in oxygen levels in COPD patients. They found no significant difference in the lowest oxygen saturation recorded during the two tests [12].

Hajare RB et al. investigated the relationship between 6MWD and spirometry parameters in 50 stable COPD patients. They performed spirometry tests measuring FEV₁, FVC, and FEV₁/FVC finding a significant positive correlation between the percent predicted 6MWD and these parameters [13]. Similarly, Chandra et al. studied the correlation between 6MWT results and spirometric findings in 60 confirmed COPD patients. Using pre- and post-bronchodilator spirometry, they observed that 6MWD was significantly positively correlated with FEV₁, FVC, FEV₁/FVC, and peak expiratory flow rate (PEFR), highlighting the relationship between lung function and exercise capacity in COPD [14].

According to a study by Mitali et al., which evaluated the relationship between 6MWT and spirometry in 130 individuals with COPD, there was a substantial association between the two measures [15]. To measure the functional exercise capacity of 50 COPD patients, Beaumont et al. conducted a study to validate the three-minute step test (3MST) and compare the 3MST and 6MWT in stable patients with COPD. At the conclusion of the 3MST and the 6MWT, there was a significant difference in heart rate and SpO_2_ [16]. Following the 3MST, lower limb tiredness was noticeably higher. According to the study's findings, patients with stable COPD can have their functional exercise capacity estimated using the 3MST instead of the 6MWT.

In a newly published study, Roberts et al. looked at adverse events that occurred during 6MWT in a large cohort of 1136 patients with moderate to severe COPD, which showed that 2.2% of the participants under investigation experienced an adverse event during the 6MWT, according to the findings. Palpitations, chest tightness, chest pain, and dizziness were the most frequent side effects. There were no noteworthy cases of morbidity or death noted. Given the significantly shorter test duration, a 2MWT would probably lower the likelihood of test-related incidents, even though the exact time of the 6MWT event occurrence was not disclosed. We observed no adverse effects from the two- or six-minute walk assessments, even though the current study was not designed to evaluate safety [17].

Beyond safety considerations, the 2MWT offers additional potential benefits over the 6MWT, particularly for patients with severe COPD. Using a shorter-duration walk test could be more practical and less tiring as it provides comparable results to the 6MWT. The 2MWT’s shorter time requirement could allow for more frequent repetitions, which would be useful for interim evaluation of treatment effects or for fine-tuning the optimal oxygen flow rate during physical activity. The 2MWT may also be useful in determining the right level of intensity for a training program that involves walking. More research is needed to determine if the 2MWT is as good as the 6MWT at determining training intensities based on walking speed. In clinical settings, the 2MWT could serve as a quicker assessment tool, saving time in busy daily clinical routines.

Limitations

Our study is the first to assess the relationship between spirometric indices and the distance covered in the 2MWT and 6MWT among severe and very severe COPD patients. However, the study has several limitations that warrant attention. First, the sample size of 50 patients, although adequate for detecting significant associations, limits the generalizability of the results, particularly across different demographics or less severe COPD cases. Secondly, the study focused exclusively on severe and very severe COPD patients, meaning that findings may not apply to patients with mild or moderate COPD, where the correlation between walk tests and spirometric indices might differ. Additionally, the study did not account for other variables like comorbidities (e.g., cardiovascular disease, diabetes, or musculoskeletal disorders) that could affect walking performance, potentially confounding the results. Finally, the cross-sectional design limits the ability to assess changes in functional capacity or spirometric indices over time, which would be valuable in longitudinal COPD management.

## Conclusions

The 2MWT and the 6MWT show good grades of congruence and correlation when evaluating exercise-induced oxygen desaturation, a critical indicator of disease condition in patients with severe COPD. Other 6MWT indicators, including heart rate, breathing rate, and perceived effort levels, were comparable to 2MWT in individuals with severe COPD. The 2MWT is easy to use and practical; these patients tolerated it well. As a result, the 2MWD might be more significant than previously thought, and there was a significant correlation between spirometric parameters and 2MWT among severe COPD patients. Hence, we conclude that the 2MWT can be used as a simple, reliable, and effective alternative tool for the assessment of prognosis in severe COPD patients.

## Appendices

**Table 6:** Questionnaire items

Section	Question/Parameter	Response Type/Options
Sociodemographic Details	Name	Open-ended
	Age	Open-ended
	Sex	Male/Female
	Address	Open-ended
	Place of Residence	Rural/Semi-Urban/Urban
	Hospital Number	Open-ended
Clinical History	Do you experience cough?	Yes/No
	Do you experience breathlessness?	Yes/No
	Other Symptoms	Open-ended
Smoking and Addiction History	Smoking type (e.g., Cigarette, Beedi)	Open-ended
	Number of cigarettes/beedis smoked per day	Open-ended
	Duration of smoking	Open-ended
	Smoking Index	Open-ended
Symptom Scales	CAT Score	Open-ended
	MMRC Grade	Open-ended
Occupational History	Occupation Details	Open-ended
Exacerbation History	History of previous exacerbations	Yes/No
	Number of exacerbations	Open-ended
	History of hospital admissions due to exacerbations	Yes/No
	Number of hospitalizations	Open-ended
Examination	General Examination	Pallor, Icterus, Cyanosis, Clubbing, Lymphadenopathy, Pedal Edema (Yes/No)
	Vitals	Pulse (beats/min), Blood Pressure (mmHg), Respiratory Rate (breaths/min)
	Type of Breathing	Abdominothoracic/Thoracoabdominal
	Upper Respiratory System Examination	Nasal Cavity, Oral Cavity, Sinuses, Pharynx (Open-ended observations)
	Lower Respiratory Tract Examination	Inspection, Palpation, Percussion, Auscultation (Open-ended observations)
	Cardiovascular System	Open-ended
	Abdominal Examination	Open-ended
	Central Nervous System	Open-ended
COPD Assessment	Known COPD	Yes/No
	Duration of COPD	Open-ended
	Currently on Treatment	Yes/No
	Duration of Treatment	Open-ended
	Type of Inhaler	MDI/DPI
	Drugs Used	Open-ended
Spirometry Values	Pre- and Post-Test Values	FEV₁, FVC, FEV₁/FVC, PEFR, FEV 25-75%
	Reversibility	Open-ended
	GOLD Staging	Open-ended
Walk Test Assessment	6-Minute Walk Test	Vitals Pre-Test: BP, RR, HR, SpO₂
		Vitals Post-Test: BP, RR, HR, SpO₂
		Distance Covered (Meters)
	2-Minute Walk Test	Vitals Pre-Test: BP, RR, HR, SpO₂
		Vitals Post-Test: BP, RR, HR, SpO₂
		Distance Covered (Meters)

**Table 7:** Vital test

	6MWT		2MWT	
VITALS	Prior to test	After test	Prior to test	After test
BP (mmHg)				
RR (/mt)				
HR (b/mt)				
SPO2 (%)				
Distance Covered (metres)				
